# Percutaneous laser ablation vs. radical nephroureterectomy: a comparative study on renal pelvic tumors

**DOI:** 10.1007/s11255-025-04395-4

**Published:** 2025-02-04

**Authors:** Gao Li, Yuanhao Chen, Xin Zhang

**Affiliations:** Department of Urology, Beijing Chao-Yang Hospital Affiliated to Capital Medical University, No. 5 Jing Yuan Road, Shi Jing Shan District, Beijing, 100043 China

**Keywords:** Renal pelvis tumor, Percutaneous laser ablation, Percutaneous endoscopic surgery, Radical nephroureterectomy

## Abstract

**Purpose:**

To compare the safety and efficacy of percutaneous laser ablation and radical nephroureterectomy for renal pelvic tumors.

**Methods:**

This prospective cohort study included 30 patients diagnosed with renal pelvic tumors who met the established selection criteria. The cohort was divided into two groups: Group I, consisting of 15 patients who underwent percutaneous laser ablation, and Group II, comprising 15 patients who received radical nephroureterectomy. Comprehensive data collection encompassed demographic information, intraoperative and postoperative outcomes, and disease-free survival.

**Results:**

The analysis revealed that percutaneous laser ablation offered modest benefits over radical nephroureterectomy in terms of reduced surgical duration (*P* < 0.01) and shorter hospital stays (*P* = 0.03). However, in evaluating long-term oncologic outcomes, percutaneous laser ablation did not achieve parity with radical nephroureterectomy. Although the differences in long-term outcomes were not statistically significant (HR: 0.48; 95% CI 0.05–4.92, *P* = 0.54), radical nephroureterectomy exhibited a slight advantage in disease-free survival.

**Conclusion:**

Percutaneous laser ablation presents a safe and effective, less invasive treatment alternative, rendering it a feasible option for patients who are either unable or unwilling to undergo radical nephroureterectomy due to comorbid conditions or personal preferences.

## Introduction

Upper urinary tract urothelial carcinoma (UTUC) represents a significant global health concern due to its life-threatening nature. In Western countries, the estimated annual incidence rate of UTUC is approximately 1–2 cases per 100,000 individuals [1]. Notably, the incidence of urothelial tumors in the upper tract continues to rise [2, 3]. The standard treatment protocol for UTUC involves radical nephroureterectomy (RNU), which includes the excision of the ipsilateral ureteral orifice. Over the past few decades, the increased utilization of computed tomography scans and urine biomarker screening tests has facilitated the earlier diagnosis of UTUC in a growing number of patients.Organ-sparing surgery, also referred to as precise surgery, has undergone rapid development, significantly transforming the conceptual framework of surgeons [4]. Recently, urologists have been exploring the application of novel treatments and technologies to benefit patients diagnosed with renal pelvic urothelial cell carcinoma [5, 6]. Kidney-sparing surgery (KSS) represents a conservative therapeutic strategy that predominantly encompasses flexible ureteroscopy and percutaneous endoscopic surgery(PC,Percutaneous laser ablation) [7]. According to European Association of Urology guidelines, KSS is applicable to select patients presenting with localized early-stage renal pelvic transitional cell carcinoma (TCC) and those possessing a solitary or functionally solitary kidney [8]. The KSS strategy has been demonstrated to maintain renal function in patients over the long term without adversely affecting oncologic outcomes. Consequently, patients benefit from the preservation of renal function and the prevention of related comorbidities [9]. These advancements hold particular significance for octogenarians [10]. In contrast, other studies suggest that oncologic outcomes in the endoscopic group may be inferior to those in the nephroureterectomy group. However, this conclusion requires further data validation [11]. The inconsistency in findings may be due to factors such as small sample sizes, study design limitations, and potential biases introduced during the research process. In addition, there is currently a lack of available studies focusing on the Chinese population. The question of whether percutaneous laser ablation is less invasive yet potentially compromises oncologic outcomes compared to radical nephroureterectomy for renal pelvic tumors remains unresolved. To address this issue, we have designed a prospective cohort study aimed at comparing the safety and oncologic outcomes of percutaneous laser ablation and radical nephroureterectomy.

## Materials and methods

### Selection of patients

In this prospective cohort study, a total of 35 patients diagnosed with a renal pelvic tumor were consecutively enrolled at Beijing Chaoyang Hospital between June 2017 and June 2020. The inclusion criteria encompassed the presence of a unifocal renal pelvic tumor, absence of infiltrative lesions on computed tomography, a clinical TNM stage of less than T2N0M0, and the provision of informed consent for rigorous postoperative follow-up. Patients were excluded if postoperative pathology did not confirm urothelial cell carcinoma, if they had multifocal renal pelvic tumors, or if they presented with concurrent ureteral or bladder tumors. Five patients were excluded based on the study's exclusion criteria, resulting in a final cohort of 30 patients. Data collection encompassed patient demographics, creatinine levels, glomerular filtration rate (GFR), and the assessment of comorbidities prior to surgery. Of these patients, 15 underwent percutaneous endoscopic laser ablation and were designated as Group I (PC group). The study encompassed eight patients who declined nephrectomy- five of whom had diabetes and three exhibited elevated blood creatinine levels. In addition, one patient presented with a congenital solitary kidney, while two others had a functional solitary kidney with atrophy. Four patients had small pelvic tumors (< 2 cm) and were at a low clinical stage. The remaining 15 patients, diagnosed with renal pelvic urothelial carcinoma (UCC) and who underwent radical nephroureterectomy, were designated as Group II (RNU group).

All patients underwent a ureteroscopic examination as part of their preoperative evaluation to assess the ureter and confirm the presence of the tumor.Preoperative computed tomography urography was performed to rule out the presence of tumors in the ureter, and this was further confirmed by intraoperative ureteroscopic examination during the procedure. Strictly adhering to the European Association of Urology guidelines, the tumor grading, tumor size, and tumor staging of these patients were evaluated.

Data collected included tumor grade, tumor size, and laterality. Tumor size was assessed clinically using computed tomography, while tumor stage was determined pathologically through cold cup biopsies, as well as intraoperative and postoperative outcomes, such as the duration of surgery,estimated blood loss, the length of hospital stay, etc.

### Surgery procedure

**Fig. 1:**
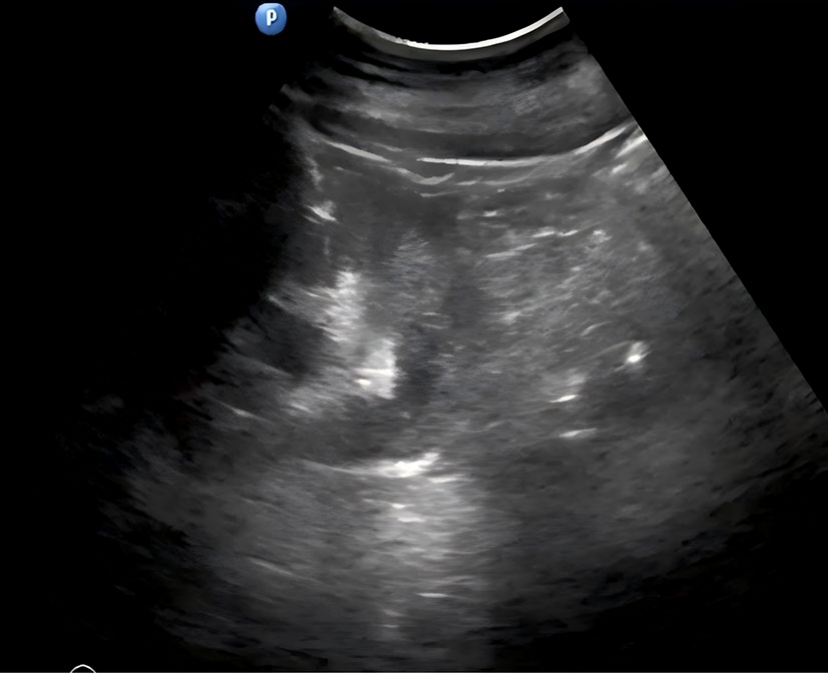
Ultrasound image of percutaneous renal puncture. Ultrasound image of establishing F18 percutaneous access to monitor the puncture route with the assistance of one step balloon dilation

In Group I, a cohort of 15 patients underwent treatment via percutaneous endoscopic laser ablation while positioned prone. The standard percutaneous approach for managing renal pelvic tumors was employed, as referenced in [12]. Ultrasound guidance facilitated the establishment of percutaneous access, as depicted in Fig. 1. The most directly accessible posterior calyx was punctured and subsequently dilated to a 24 French aperture using a balloon (Bard Corp N30, USA). Cold cup biopsies were obtained and submitted for histopathological examination prior to vaporization. The tumor was ablated using a 1470 nm laser (Qizhi Corp, China) with a cutting power setting of 120 W and a coagulative power setting of 80 W under percutaneous nephroscopy (Wolf Corp, Germany). Following the surgical procedure, a 6F double-J stent and a 14F nephrostomy tube were inserted. Subsequently, within 1 week post-surgery, a perfusion of 50-mg mitomycin C in 50-ml saline was administered via the nephrostomy tube. The nephrostomy tube was removed prior to patient discharge, while the double-J stent was extracted 2 to 4 weeks postoperatively.

In Group II, 15 patients underwent laparoscopic radical nephroureterectomy; however, two of these patients required conversion to open surgery due to severe adhesions.

### Follow-up protocol

Following the surgical procedure, all patients underwent a scheduled cystoscopy at 3 months post-surgery, subsequently every six months during the first two years, and annually thereafter. At the initial follow-up visit, three months post-surgery, assessments of creatinine levels, GFR, and surgical complications, categorized according to the Clavien–Dindo classification, were conducted. Disease-free survival (DFS) for the patients was defined as the duration of survival without any local disease recurrence, metastasis, or death from any cause.

### Statistical analysis

Statistical analysis was performed with STATA/ IC16.1 (Stata Corp, College Station, TX, USA). Continuous data with normal distribution are shown as the mean ± standard deviation. Skewed continuous data are shown as median (interquartile ranges, IQR), Comparisons normally distributed continuous data between the two groups were performed using the *t* test. Non-normally distributed continuous data comparisons were performed using the Mann–Whitney *U* test. Kaplan–Meier survival curves were presented after being adjusted for tumor stage and grade. A log-rank test was used to compared treatment groups after adjusting tumor stage and grade. Cox Proportional Hazard regression model was used to adjust unbalanced confounders and compare the hazard ratio (HR) between groups; proportion hazard assumption was tested as well. The level of statistical significance was defined as *P* < 0.05.

## Results

### Baseline characteristics

Among the cohort of patients diagnosed with renal pelvic carcinoma, there were 21 males and 9 females, with a mean age of 65.5 ± 6.1 years. Statistical analysis revealed no significant differences in age, sex, body mass index (BMI), Charlson comorbidity scores, or pre-surgical comorbidities between the two groups. In Group I, three patients presented with anatomic or functional solar kidneys, whereas no patients in Group II exhibited this condition; however, this difference was not statistically significant, as detailed in Table 1. Furthermore, the baseline creatinine levels and the creatinine levels measured three months post-surgery showed no statistically significant difference between the two groups, and there was also no difference in GFR before surgery and three months after surgery, as presented in Table 2.

**Table 1:** Patients characteristics and tumor characteristics of groups I and II

	Group I (PC)	Group II (RNU)	*P* values
	*N* = 15	*N* = 15	
Patients characteristics			
Age, mean (SD)	65.7 (5.9)	65.4 (6.4)	0.91
Gender			0.69
Female	5 (33%)	4 (27%)	
Male	10 (67%)	11 (73%)	
BMI, mean (SD)	25.4 (2.9)	25.2 (3.8)	0.91
Solar Kidney			0.07
No	12 (80%)	15 (100%)	
Yes	3 (20%)	0 (0%)	
Charlson score, mean (SD)	2.9 (1.2)	2.7 (1.0)	0.62
Hypertension			0.71
No	9 (60%)	8 (53%)	
Yes	6 (40%)	7 (47%)	
Diabetes mellitus			0.07
No	10 (67%)	14 (93%)	
Yes	5 (33%)	1 (7%)	
Chronic obstructive pulmonary disease			0.31
No	15 (100%)	14 (93%)	
Yes	0 (0%)	1 (7%)	
Myocardial infarction			1.00
No	13 (87%)	13 (87%)	
Yes	2 (13%)	2 (13%)	
Cardiovascular disease			0.41
No	12 (80%)	10 (67%)	
Yes	3 (20%)	5 (33%)	
Stroke			0.14
No	14 (93%)	11 (73%)	
Yes	1 (7%)	4 (27%)	
Peripheral vascular disease			0.31
No	15 (100%)	14 (93%)	
Yes	0 (0%)	1 (7%)	
Ulcer			1.00
No	14 (93%)	14 (93%)	
Yes	1 (7%)	1 (7%)	
Tumor characteristics			
Tumor side			1.00
Left	8 (53%)	8 (53%)	
Right	7 (47%)	7 (47%)	
pT−stage			0.59
pTa	8 (53%)	7 (47%)	
pT1	7 (47%)	7 (47%)	
pT2	0 (0%)	1 (7%)	
Grade			0.71
Low grade	9 (60%)	8 (53%)	
High grade	6 (40%)	7 (47%)	
Clinical tumor size(cm), mean (SD)	1.8 (0.7)	3.1 (0.7)	<0.01*

*Indicates *P* < 0.05. *PC* Percutaneous laser ablation, *RNU* Radical nephroureterectomy, *SD* Standard deviation

**Table 2:** Surgery characteristics of groups I and II

	Group I (PC)	Group II (RNU)	*P* values
	*N* = 15	*N* = 15	
Surgery time(h), median (IQR)	95.0 (79.0, 145.0)	250.0 (190.0, 285.0)	< 0.01*
Estimated blood loss (ml), median (IQR)	80.0 (60.0, 120.0)	110.0 (90.0, 120.0)	0.20
Hospital stays (days), median (IQR)	7.0 (5.0, 9.0)	9.0 (7.0, 12.0)	0.03*
Clavien comorbidity grade			0.72
Grade 0	12 (80%)	12 (80%)	
Grade I	1 (7%)	2 (13%)	
Grade II	2 (13%)	1 (7%)	
Baseline creatinine (μmol/L), median (IQR)	80.0 (75.0, 99.0)	87.0 (72.0, 94.0)	0.84
Follow-up creatinine (μmol/L), median (IQR)	85.0 (80.0, 90.0)	86.0 (77.0, 98.0)	0.97
Baseline GFR (ml/min/1.73m^2^), mean (SD)	76.6(21.1)	73.8(20.2)	0.71
Follow-up GFR (ml/min/1.73m^2^), mean (SD)	71.2(19.1)	72.4(21.1)	0.87

*Indicates *P* < 0.05. *PC* Percutaneous laser ablation, *RNU* Radical nephroureterectomy, *IQR* Interquartile range, *SD* Standard deviation, *GFR* Glomerular filtration rate

### Pathologic results

The tumor sizes varied from 0.9 cm to 4.5 cm. The mean tumor size in Group I was 1.8 ± 0.7 cm, whereas in Group II, the mean tumor size was 3.1 ± 0.7 cm, which was significantly larger than that of Group I (*P* < 0.01). This finding suggests that patients with larger tumors are more likely to undergo RNU rather than PC. The differences in pathologic tumor grade and stage between the two groups were not statistically significant, as presented in Table 2.

### Procedural characteristics

Group I demonstrated a shorter operative time and reduced duration of hospitalization compared to Group II. Specifically, in Group I, the median operative time was 95 min (IQR 79–145 min), the median estimated blood loss was 80 ml (IQR 60–120 ml), and the median hospital stay was 7 days (IQR 5–9 days), as detailed in Table 2. Conversely, in Group II, the median operative time was 250 min (IQR 190–285 min), the median estimated blood loss was 110 ml (IQR 90–120 ml), and the median hospital stay was 9 days (IQR 7–12 days). No statistically significant difference in blood loss was observed between the groups (*P* = 0.2). However, the median operation time was significantly shorter in Group I compared to Group II (*P* < 0.001), and the median hospital stay was also notably shorter in Group I than in Group II (*P* = 0.03). In addition, no significant difference was detected in the Clavien surgery comorbidity grade between the two groups, as detailed in Table 2.

### Postoperative outcome

**Fig. 2:**
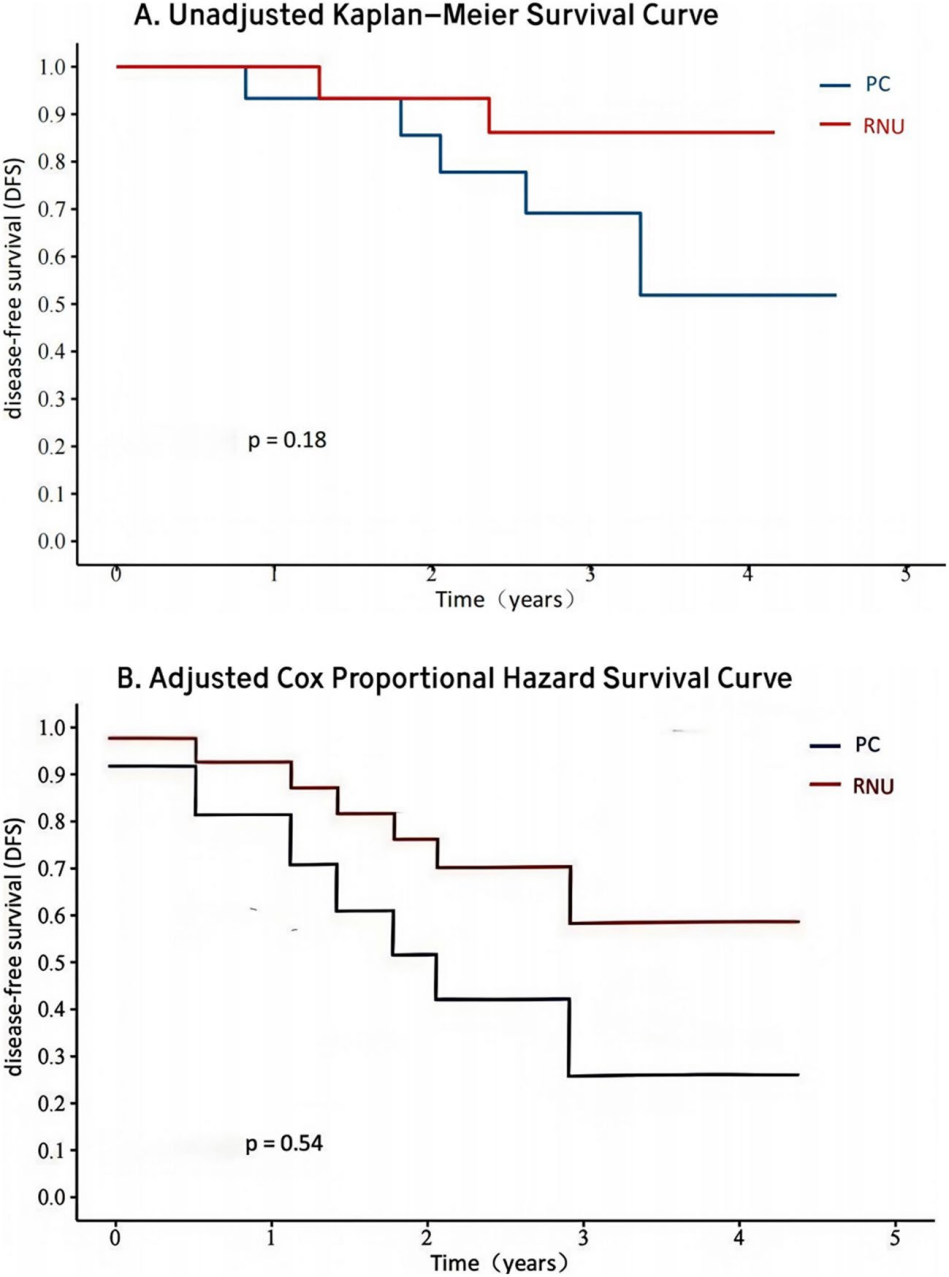
Unadjusted Kaplan–Meier and Adjusted Cox Proportional Hazards Survival Curves for percutaneous laser ablation and radical nephroureterectomy Cohorts. **A** Unadjusted Kaplan–Meier survival curves stratified by procedure with log-rank test (*P* = 0.18). **B** Adjusted Cox Proportional Hazards Survival Curves, including tumor size, grade, and stage, stratified by procedure with Cox Proportional Hazards regression model (HR: 0.48; 95% CI 0.05–4.92, *P* = 0.54)

Postoperatively, the follow-up period extended from 10 to 54 months, with an average duration of 34 months. Within this period, Group I experienced five adverse events: two patients succumbed to cardiovascular comorbidities, two patients experienced local recurrence and subsequently underwent RNU as part of their treatment, and one developed systemic bone metastasis. In contrast, Group II reported two fatalities during follow-up—one due to stroke and the other to myocardial infarction-without any occurrence of local or systemic metastasis. Throughout the entire follow-up duration, no evidence of tract seeding was detected, as confirmed by routine enhanced computed tomography scans. Although the unadjusted Kaplan–Meier curve suggests a trend indicating that Group I experienced more events than Group II, the log-rank test did not reveal a statistically significant difference between the two groups (*P* = 0.18) (shown in Fig. 2A). After adjusting for tumor size, grade, and stage using the Cox Proportional Hazards regression model, the HR for DFS was 0.48 (95% CI 0.05–4.92, *P* = 0.54), as depicted in the Cox model-predicted survival curve (shown in Fig. 2B). Although there appeared to be a trend suggesting that the hazard for Group II was lower than that for Group I, this observation did not reach statistical significance, likely due to the small sample size.

## Discussion

Our preliminary study showed that percutaneous laser ablation had much less surgery time and hospital stay than radical nephroureterectomy with similar renal function during the follow-up. The HR of DFS in standard radical nephroureterectomy (group II) was lower than that in patients who were treated with percutaneous laser ablation laser treatment (group I), but it is not statistically significant. This may be due to the small sample size, which lacks sufficient statistical power to detect significant differences. Both the unadjusted K–M survival curve and the Cox regression-adjusted survival curve suggest that percutaneous laser ablation did not match the oncologic outcomes of radical nephroureterectomy, but further data validation is required. But from the preliminary data, we can calculate that to detect the statistically significance between the two group we need at least 72 events with 5 years follow-up time. The total sample size should be more than 200 patients. It is unrealistic for a single center to recruit such a large number of pelvic tumor patients. This study suggests that endoscopic laser treatment is significantly less invasive, with less blood loss and shorter hospital stays, but it may have an impact on oncologic outcomes. Given the small sample size, further research is essential to confirm these findings.

Some of the patients in this study did not conform to standard current guidelines regarding tumor size, grading, and stage. This was unavoidable for urologists who treated these patients with a solitary kidney, concurrent bilateral malignancy, renal insufficiency, concomitant medical conditions, personal concerns, or octogenarians. These conditions were prohibitive for a major operation, such as nephroureterectomy [13]. In certain cases, particularly when radical nephroureterectomy is not feasible, percutaneous laser ablation remains a safe and effective treatment option, especially for patients with compromised kidney function or those who refuse nephrectomy, considering the limited alternatives available. The European Association of Urology guideline has already approved recommendations to renal pelvis carcinoma patients with carefully selected characteristics [14].

Retrograde flexible ureteroscopy with laser treatment is a choice for treatment of some renal tumor carcinomas [2]. Unfortunately, 500-μm lasers cannot be applied via a ureteroscope with a small lumen, which is much more effective in tissue vaporization [15]. Therefore, the 200 μm holmium laser applied in flexible ureteroscopy is not sufficiently effective to carry out tumor vaporization for larger tumors [4]. In our clinical practice, we first carried out this nephron-sparing procedure in patients with special conditions, such as those with a congenital solitary kidney or functional solitary kidney, or those unwilling to receive radical nephrectomy. Local re-occurrence or the metastasis rate is still high during postoperative follow-up in these patients. In recent years, using evidence-based medicine, many guidelines have recommended strict criteria for kidney-sparing surgery in patients with UTUC [16]. These include a unifocal tumor, a tumor < 1 cm, a low-grade tumor, no evidence of an infiltrative lesion on computed tomography, and understanding of strict follow-up after surgery [6]. A laser should usually be used for endoscopic treatment and flexible ureteroscopy is preferred for rigid ureteroscopy, regardless of whether the tumor is in the renal pelvis or in the distal, mid, or proximal ureter [17, 18].

In previous reports, most of these procedures were finished by laser via flexible ureteroscopy. This retrograde pathway is often affected by the condition of the ureter and a large portion of patients require placement of the ureteral sheath before a second-stage treatment [9]. When using a flexible ureteroscope, several factors, such as a narrow and confined space, make tumor ablation difficult and slow, and increase the chance of infection. In addition, tumors are sometimes not accessible, especially for tumors at the inferior calices [10]. With wide application of PC, it is no longer only suitable for kidney stone treatment. The percutaneous approach has many advantages, including a wide lumen space, sufficient liquid outflow, and accessibility to target calices. PC can provide a better view, and more importantly, it can lower renal pelvic pressure by free liquid outflow and significantly reduce occurrence of postoperative sepsis compared with flexible ureteroscopy [19]. In addition, under percutaneous access, a wider aperture and more powerful laser for vaporization can be applied to achieve a more effective ablation result [12]. Furthermore, bladder chemotherapy can also be applied via a renal fistula tube after this procedure. After removal of the fistula, bladder instillation therapy can be performed when a ureteral double-J stent remains [13]. However, this is still controversy regarding effectiveness of bladder instillation in tumor control.

There have been no previous reports on patients with UCC who refused radical nephroureterectomy, either because of personal concerns or because of a solitary function kidney, and who had percutaneous endoscopy laser ablation instead. In our study, we compared patients with renal pelvic carcinoma who were treated with laser ablation under percutaneous endoscopy and those who received standard radical nephroureterectomy. To the best of our knowledge, this study is the first prospective cohort study to compare these treatments in Chinese patients with UTUC.

Several limitations of this study warrant consideration. First, the sample size was limited to 30 patients, which may compromise the robustness of the statistical analyses, particularly since some treatment comparisons did not achieve statistical significance. Although percutaneous laser ablation demonstrated benefits in terms of reduced surgical duration and shorter hospital stays, the small sample size may not adequately capture the true differences between the two treatment modalities. Second, the follow-up period was relatively brief, with an average duration of 34 months. While no local or systemic metastasis was detected during this period, short-term data are insufficient to fully assess the long-term effects of the treatments. Given the potential for renal pelvic carcinoma to exhibit a prolonged latency period, extended follow-up studies are necessary to more accurately evaluate the long-term outcomes, especially concerning recurrence and metastasis rates. Lastly, the study did not comprehensively account for other potential influencing factors, such as patients’ quality of life, which could significantly affect overall treatment outcomes. Future studies should incorporate additional clinical indicators, especially quality of life measures, to more comprehensively evaluate the overall efficacy of the two treatment options.

## Conclusion

Percutaneous laser ablation for renal pelvic tumors presents modest advantages over radical nephroureterectomy concerning surgical duration and hospital stay, characterized by reduced operation times and shorter hospitalizations. However, with respect to long-term oncologic outcomes, percutaneous laser ablation does not achieve results equivalent to those of radical nephroureterectomy. Although the disparity in long-term outcomes is not pronounced, radical nephroureterectomy demonstrates a slight superiority in disease-free survival. Nevertheless, percutaneous laser ablation offers a less invasive alternative, rendering it a viable treatment option for patients who are either unable or unwilling to undergo radical nephroureterectomy due to comorbidities or personal preferences. This study substantiates that percutaneous laser ablation can serve as a feasible alternative for such patients, although further research with larger sample sizes and extended follow-up periods is essential to more comprehensively assess its long-term efficacy.

## Data Availability

The data that support the findings of this study are not publicly available because they contain information that could compromise the privacy of research participants, but are available from the corresponding author (Xin Zhang) upon reasonable request. E-mail:18201636330@163.com.

## Abbreviations

PC: Endoscopic surgery
RNU: Radical nephroureterectomy
DFS: Disease-free survival
UCC: Urothelial cell carcinoma
HR: Hazard ratio
CI: Confidence interval
BMI: Body mass index
UTUC: Upper urinary tract urothelial carcinoma
KSS: Kidney-sparing surgery
TCC: Transitional cell carcinoma
GFR: Glomerular filtration rate
IQR: Interquartile ranges
SD: Standard deviation
